# Measuring the impact of methodological research at the medical research council clinical trials unit hub for trials methodology research

**DOI:** 10.1186/1745-6215-14-S1-O126

**Published:** 2013-11-29

**Authors:** Valerie Brueton, Claire Vale, Rachel Jinks, Babak Oskooei, Jayne Tierney

**Affiliations:** 1 MRC Clinical Trials Unit Hub for Trials Methodology Research, London, UK

## Background

Evidence of research impact is increasingly requested by research funders. This commonly relies on citation analysis; however other indicators may be more informative.

## Methods

We aimed to measure multiple indicators of impact for methodological research projects conducted or completed at the UK Medical Research Council Clinical Trials Unit (CTU) from 2009-2012. Publication citation rates were obtained using ISI Web of Science. Email queries generated by these projects were recorded retrospectively. Semi-structured interviews were conducted with CTU methodologists to obtain further data on the impact of methods. Interviews and email content were analysed, along with a descriptive analysis of citations.

## Results

Preliminary results are based on 38 publications from 2009-2010 and 21 research projects. Seven publications had >10 citations/year. Approximately 70% of these citations were in primary research publications in both general and specialised medical journals. Six methodological projects generated 194 email queries from 170 individuals across 23 countries. The 13 interviews revealed impacts such as the application of our methods by other researchers, further development and dissemination of statistical software, establishment of new collaborations and uptake of research findings in trial guidelines. Updated results will be presented.

## Conclusions

Citation rates have indicated which projects and journals generate most interest and email queries have shown which methodological developments are in widespread use. Through the interviews we picked up a range of less obvious but important impacts. These results will be used to monitor the uptake and impact of our methodological research, providing a framework that others might use.

